# Association between dietary patterns and the risk of all-cause mortality among old adults with obstructive sleep apnea

**DOI:** 10.1186/s12877-024-05126-7

**Published:** 2024-07-02

**Authors:** Wei Zhao, Lu Gao, Zhiyuan Wu, Mingzhao Qin

**Affiliations:** 1 Department of Geriatrics, Beijing Tongren Hospital, Capital Medical University, Beijing, 100730 People’s Republic of China; 2 Harvard T.H. Chan School of Public Health, 655 Huntington Ave, Boston, MA 02115 United States

**Keywords:** aMED, HEI-2015, AHEI-2010, Obstructive sleep apnea, All-cause mortality

## Abstract

**Background:**

Obstructive sleep apnea (OSA) was associated with the increased cardiovascular events and all-cause mortality. And anti-inflammatory dietary has potential to improve the prognosis of OSA. This study aimed to investigate the association of anti-inflammatory dietary patterns with all-cause mortality among individuals with OSA.

**Methods:**

This retrospective cohort study involved 1522 older adults with OSA from 2005 to 2008 in the National Health and Nutrition Examinations Survey (NHANES). Mortality status was determined by routine follow-up through December 31, 2019, using the National Death Index. Anti-inflammatory dietary patterns included Alternate Mediterranean Diet Score (aMED), Healthy Eating Index-2015 (HEI-2015), and Alternate Healthy Eating Index-2010 (AHEI-2010). Weighted Cox proportional hazard regression models were performed to investigate the association between anti-inflammatory dietary pattern and all-cause mortality.

**Results:**

After a median follow-up of 131 months, 604 participants were recorded all-cause mortality. The mean age of OSA patients was 68.99 years old, of whom 859 were male (52.34%). Higher adherence of aMED (HR = 0.61, 95%CI: 0.48 to 0.78) and HEI-2015 (HR = 0.75, 95%CI: 0.60 to 0.95) were associated with lower all-cause mortality risk in the elderly with OSA. Conversely, no association was found between AHEI-2010 dietary pattern and all-cause mortality in individuals with OSA. In the component analysis of aMED, it was found that a higher intake of vegetables and olive oil potentially contributes to the reduction all-cause mortality risk in the elderly with OSA (HR = 0.60, 95%CI: 0.48 to 0.76; HR = 0.67, 95%CI: 0.63 to 0.71).

**Conclusion:**

Higher adherence to the aMED and the HEI-2015 was associated with a lower risk of all-cause mortality in OSA. Future interventions in the elderly with OSA should considering adopting anti-inflammatory dietary patterns.

## Background

Obstructive sleep apnea (OSA) is a common chronic disease characterized by recurrent episodes of upper airway obstruction or reduced respiratory amplitude, causing low oxygen levels in the arteries, increased carbon dioxide levels, and brief awakenings during sleep, resulting in disrupted sleep [1]. The prevalence of OSA ranges from 27 to 80% in individuals aged ≥ 60 years [2]. As the global population aging, the prevalence of OSA is expected to rise, affecting a large portion of older individuals [3]. OSA increases the likelihood of cardiovascular (CV) events and contributes to elevated all-cause mortality, mainly due to oxidative stress induced by nocturnal intermittent hypoxia [4, 5]. Severe OSA is an independent risk factor for both all-cause and CV mortalities [6]. Patients with OSA who show high OSA-specific hypoxic burden are at a heightened risk of all-cause mortality [7]. Therefore, reducing systemic oxidative stress levels may be beneficial in reducing the mortality risk in OSA patients.

Mediterranean diet has been shown to have anti-inflammatory and antioxidant properties in OSA, and can reduce the incidence and mortality rate of major CV events [8, 9]. Diets plays a crucial role in modulating inflammation, and a healthier, particularly anti-inflammatory diet, may enhance the prognosis of OSA [10, 11]. The Alternate Mediterranean Diet Score (aMED), Healthy Eating Index-2015 (HEI-2015), and Alternate Healthy Eating Index-2010 (AHEI-2010) are international recognized assessment tools commonly used to evaluate dietary quality, and they were related to circulating inflammation and oxidative stress [12–14]. A meta-analysis indicated that the Mediterranean diet can decrease all-cause mortality [15]. Another study found that higher adherence to the 2015–2020 Dietary Guidelines for Americans was associated with a reduced risk of CVD and all-cause mortality [16]. Adherence to AHEI-2010 was associated with a lower risk of chronic disease mortality in an Asian population [17]. Studies reported that greater adherence to these healthy dietary patterns was linked to a reduced mortality risk in the general population [18, 19]. We speculated that anti-inflammatory dietary patterns may also be beneficial in OSA patients.

However, there is insufficient research on the impact of anti-inflammation dietary patterns on mortality risk among the elderly with OSA. We aimed to explore the association between anti-inflammation dietary patterns and all-cause mortality risk in the elderly with OSA, and further explore this association in different subgroups.

## Methods

### Study design and participants

Data in this retrospective cohort study were extracted from the National Health and Nutrition Examinations Survey (NHANES). The NHANES is a program designed to assess the health and nutritional status of adults and children in the US [20]. The study protocol was approved by the National Center for Health Statistics (NCHS) Research Ethics Review Board and all participants have signed informed consents. The requirement of ethical approval for this was waived by the Institutional Review Board of Beijing Tongren Hospital, Capital Medical University, because the data was accessed from NHANES (a publicly available database). All methods were performed in accordance with the relevant guidelines and regulations.

**Fig. 1:**
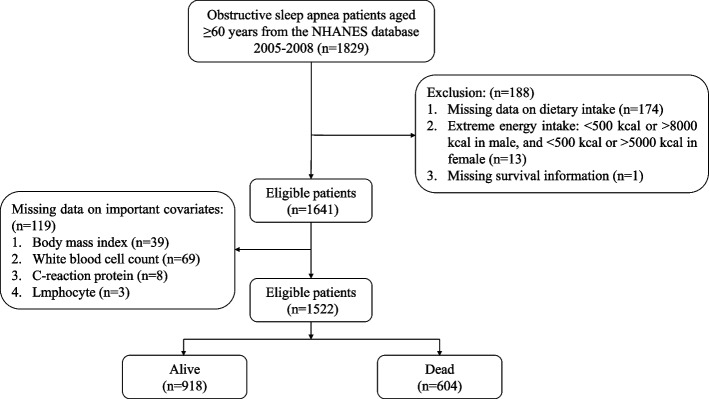
Flow chart of participants selection

Participants with OSA aged 60 years and above were included from two cycles (2005–2006, 2007–2008). OSA is diagnosed according to Healthy People 2020 [21] and defined as meeting any of the following conditions: (1) doctor diagnosed sleep apnea; (2) snoring 3 or more nights per week; (3) snoring, gasping or stopping breathing 3 or more nights per week; (4) feeling excessively sleepy during the day 16–30 times per month despite sleeping around 7 or more hours per night on weekdays or work nights. Participants with implausible energy intake were excluded. Then, participants were excluded with missing dietary intake information and survival information. Participants with missing data on significant covariates (body mass index (BMI), white blood cell (WBC), C-reactive protein (CRP), and lymphocytes were also excluded. Figure 1 shows the complete selection process.

### Outcome ascertainment

Mortality information of OSA patients was linked with the NHANES datasets. All data in this study were publicly available (https://www.cdc.gov/nchs/data-linkage/mortality.htm). All-cause mortality was determined by the National Death Index before 31 December 2019. Follow-up time was calculated from the date of interview to the date of death or the end of follow-up.

### Dietary intake

Dietary intake information was obtained from 24-h dietary recalls at baseline, facilitated by a trained interviewer and employing a Computer Assisted Dietary Interview and Automated Multiples Pass Method. In order to ensure precision in estimating portion sizes, measuring utensils were employed. The data encompassed food and beverage intake within the previous 24 h and was subsequently uploaded into the US Department of Agriculture’s Food and Nutrient Database for Dietary Studies and the Food Patterns Equivalence Database for comprehensive evaluation of nutrient composition and food patterns, respectively.

### Anti-inflammatory dietary assessment

aMED: The aMED score (total score = 18) is derived from a score of "0", "1" or "2" for each of the nine food groups (vegetables, legumes, fruits, nuts, whole grains, red and processed meats, fish, alcohol, and olive oil), with higher scores indicating better adherence to the Med pattern [22, 23].

AHEI-2010: AHEI-2010 consists of eleven components, with six encouraging (vegetables, fruits, whole grains, nuts/legumes, omega-3 fatty acids and polyunsaturated fatty acids) and four limiting (sugar-sweetened beverages and fruit juices, red and processed meat, trans fatty acids and sodium). In AHEI-2010 the components are scored from 0 to 10 and the total score from 0 to 110, with higher scores indicates higher quality diet [24, 25].

HEI-2015: HEI-2015 is a measure for assessing dietary quality with nine encouraging (total fruit, whole fruit, total vegetables, greens and beans, whole grains, dairy, total protein foods, seafood and plant proteins, and the ratio of unsaturated to saturated fats) and four limiting components (refined grains, sodium, added sugars and saturated fats) [12]. These components are scored based on energy density of 1000 kcal, with the exception of fatty acids, which are the ratio of unsaturated to saturated fatty acids. Each component has a score of 0 to 5 or 0 to 10, with higher HEI scores indicate better diet quality [26]. The population ratio methods were used to calculate the mean score.

### Potential covariates

The selected covariates included age, gender, poverty income ratio (PIR), marriage, education level, BMI, physical activity, cardiovascular drugs, hypertension, diabetes, cardiovascular disease (CVD), chronic kidney disease (CKD), chronic obstructive pulmonary disease (COPD), cancer, CRP, WBC, and neutrophils. The age, gender, PIR, marriage, education level, and medication use were collected from standardized questionnaires. PIR is calculated by dividing family income by poverty guidelines (specific to family size) and the appropriate year and state. This variable is an indicator of the ratio of household income to poverty scores. BMI was obtained from physical examinations in the mobile examination center. Hypertension was defined as self-reported hypertension, or taking antihypertensive drugs, or systolic blood pressure ≥ 130 mmHg, or diastolic blood pressure ≥ 80 mmHg [27]. Diabetes was defined as self-reported diabetes, or taking hypoglycemia drugs/insulins, or hemoglobin A1c (HbA1c) ≥ 6.5%, or fasting plasma glucose ≥ 7 mmol/L [28]. Questions were used for determine each type of CVD. Participants was defined if they answered “Yes” to the questionnaire “Have you ever been told by a doctor that you had heart failure, heart attack, coronary heart disease, stroke and congestive heart failure?” [29]. CKD was defined as urinary albumin to creatinine ratio > 30 mg/g or estimated glomerular filtration rate < 60 mL/min/1.73m^2^ according to the “KDIGO 2021 Guidelines” [30]. COPD was defined as individuals who answered “Yes” to the question “Has a doctor or other health professional ever told you that you had chronic bronchitis?” or “Has a doctor or other health professional ever told you that you had emphysema?” [31]. Cancer was defined as a positive answer to the question “Have you ever been told by a doctor or other health professional that you/ had cancer or a malignancy of any kind?”. During the medical examinations, serum samples were collected and analyzed. CRP levels were determined using latex-enhanced nephelometry. The Beckman Coulter methods was employed to measure cell counts of WBC and neutrophils.

### Statistical analysis

Continuous variables were presented as mean and standard error (S.E), while categorical variables were presented as numbers and percentages. The differences in characteristics were assessed using weighted t-tests for continuous variables and Rao-Scott chi-square tests for categorical variables. All dietary intake data were weighted using the dietary day one sample weight derived from the sampling weights provided by NHANES. Potential covariates were selected using weighted univariate Cox regression analyses. Weighted univariate and multivariable Cox regression models were used to investigate the relationship between anti-inflammatory dietary and all-cause mortality in the elderly of OSA. All results were shown as hazard ratios (HRs) and 95% confidence intervals (CIs). In model 1, covariates adjusted for age, race, PIR, marriage and education. In model 2, covariates adjusted for age, race, PIR, marriage, education, BMI, physical activity, hypertension, diabetes, CVD, CKD, COPD, cancer, use of cardiovascular drugs, WBC, neutrophil and CRP. The total aMED, HEI-2015 and AHEI-2010 scores were divided into three subgroups by tertiles, and the lowest tertile of the anti-inflammatory dietary pattern were as the reference group. Subgroup analyses were performed in different gender, BMI, hypertension, diabetes, CVD, CKD, COPD and cancer subgroups. The potential nonlinear associations between anti-inflammatory dietary and all-cause mortality were also investigated by using the restricted cubic spline (RCS). All analyses were performed using R version 4.2.3 and *P* < 0.05 indicating statistical significance.

## Results

### Characteristics of participants

Totally, 1552 OSA patients were included for further analysis. Table 1 shows the characteristics of OSA patients. The mean age of OSA patients was 68.99 (± 0.23) years old, of them 859 (52.34%) were male. After a median follow-up of 131 months, 604 patients were dead. The age of OSA patients who had died was significantly older than the survived. Participants who had died were more frequently males, non-Hispanic white, with PIR ≥ 1, married or living with partner, with education level below high school, and had mild physical activity. There are statistical differences between two groups in age, race, marriage, education level, physical activity, BMI, hypertension, diabetes, CVD, CKD, COPD, cancer, use of cardiovascular drugs, WBC, neutrophil, CRP, aMED score, and HEI-2015 score (all *P* < 0.05).

**Table 1:** Characteristics of the elderly with OSA

Variables	Total (N = 1522)	Survival (N = 918)	Dead (N = 604)	Statistics	*P*
Age, years, Mean (± S.E)	68.99(± 0.23)	66.67(± 0.26)	72.84(± 0.39)	t = 12.901	< 0.001
Gender, n (%)				χ^2^ = 2.496	0.124
Male	859(52.34)	484(50.49)	375(55.42)		
Female	663(47.66)	434(49.51)	229(44.58)		
Race, n (%)				χ^2^ = 3.919	0.033
Non-Hispanic White	859(81.51)	462(80.37)	397(83.41)		
Non-Hispanic Black	267(6.90)	156(6.05)	111(8.30)		
Mexican American	236(5.15)	187(6.32)	49(3.20)		
Other Race	160(6.44)	113(7.25)	47(5.08)		
PIR, n (%)				χ^2^ = 2.427	0.110
< 1	200(6.65)	110(5.41)	90(8.70)		
≥ 1	1207(86.68)	735(88.23)	472(84.11)		
Unknown	115(6.67)	73(6.36)	42(7.19)		
Marriage, n (%)				χ^2^ = 20.780	< 0.001
Married/Living with partner	1057(71.45)	674(76.85)	383(62.48)		
Spinsterhood/Separated/Divorced/Widowed	465(28.55)	244(23.15)	221(37.52)		
Education, n (%)				χ^2^ = 22.852	< 0.001
Below High School	541(23.92)	292(17.66)	249(34.33)		
High School/GED or Equivalent	391(28.32)	231(27.98)	160(28.89)		
Above High School	590(47.76)	395(54.36)	195(36.78)		
BMI, n (%)				χ^2^ = 3.889	0.032
< 25	323(20.83)	171(18.14)	152(25.31)		
25–30	549(37.30)	333(39.15)	216(34.23)		
≥ 30	650(41.87)	414(42.72)	236(40.45)		
Physical activity, n (%)				χ^2^ = 6.066	0.004
Mild	731(42.22)	440(41.27)	291(43.78)		
Moderate/Heavy	522(37.62)	346(40.97)	176(32.05)		
Unknown	269(20.16)	132(17.76)	137(24.17)		
Smoking, n (%)				χ^2^ = 0.641	0.520
Never	665(42.85)	438(44.50)	227(40.09)		
Former	668(43.52)	373(42.55)	295(45.14)		
Now	189(13.63)	107(12.95)	82(14.77)		
Hypertension, n (%)				χ^2^ = 4.670	0.039
No	235(14.13)	164(15.99)	71(11.03)		
Yes	1287(85.87)	754(84.01)	533(88.97)		
Diabetes, n (%)				χ^2^ = 11.761	0.002
No	1035(72.70)	654(76.07)	381(67.11)		
Yes	487(27.30)	264(23.93)	223(32.89)		
Dyslipidemia, n (%)				χ^2^ = 2.586	0.118
No	212(13.30)	119(12.11)	93(15.27)		
Yes	1310(86.70)	799(87.89)	511(84.73)		
CVD, n (%)				χ^2^ = 87.887	< 0.001
No	1124(74.65)	757(83.07)	367(60.67)		
Yes	398(25.35)	161(16.93)	237(39.33)		
CKD, n (%)				χ^2^ = 85.690	< 0.001
No	1090(74.38)	750(84.02)	340(58.35)		
Yes	432(25.62)	168(15.98)	264(41.65)		
COPD, n (%)				χ^2^ = 5.569	0.025
No	1339(87.07)	839(89.46)	500(83.09)		
Yes	183(12.93)	79(10.54)	104(16.91)		
Cancer, n (%)				χ^2^ = 12.853	0.001
No	1251(81.29)	789(84.48)	462(76.00)		
Yes	271(18.71)	129(15.52)	142(24.00)		
Use of cardiovascular drugs, n (%)				χ^2^ = 45.142	< 0.001
No	1057(67.08)	709(73.88)	348(55.77)		
Yes	465(32.92)	209(26.12)	256(44.23)		
WBC, 1000/uL, Mean (± S.E)	7.16(± 0.08)	6.99(± 0.09)	7.44(± 0.14)	t = 2.894	0.007
Lymphocyte, 1000/uL, Mean (± S.E)	2.03(± 0.04)	2.04(± 0.04)	2.00(± 0.08)	t = -0.433	0.668
Neutrophil, 1000/uL, Mean (± S.E)	4.27(± 0.05)	4.11(± 0.06)	4.53(± 0.07)	t = 5.171	< 0.001
CRP, Mean (± S.E)	0.45(± 0.02)	0.39(± 0.03)	0.56(± 0.04)	t = 3.739	0.001
aMED score, n (%) -tertile				χ^2^ = 5.700	0.006
< 4	366(25.38)	196(21.96)	170(31.07)		
4–6	568(36.81)	334(36.26)	234(37.72)		
≥ 6	588(37.81)	388(41.78)	200(31.21)		
AHEI-2010 score, n (%)-tertile				χ^2^ = 0.960	0.366
< 32	500(30.81)	283(28.74)	217(34.25)		
32–40	508(33.11)	313(33.72)	195(32.09)		
≥ 40	514(36.08)	322(37.53)	192(33.66)		
HEI-2015 score, n (%)-tertile				χ^2^ = 3.227	0.053
< 47.11	507(32.36)	291(29.56)	216(37.00)		
47.11–59.43	507(33.33)	312(34.82)	195(30.84)		
≥ 59.43	508(34.31)	315(35.61)	193(32.15)		

*S.E* Standard Error, *t* Weighted t test, *χ*^*2*^ Rao-Scott Chi-square test, *OSA* Obstructive sleep apnea, *PIR* Poverty income ratio, *BIM* Body mass index, *CVD* Cardiovascular disease, *CKD* Chronic kidney disease, *COPD* Chronic obstructive pulmonary disease, *WBC* White blood cell, *CRP* C-reactive protein, *aMED* Alternate Mediterranean Diet Score, *AHEI-2010* Alternate Healthy Eating Index-2010, *HEI-2015* Healthy Eating Index-2015

### Anti-inflammatory dietary patterns and all-cause mortality

**Fig. 2:**
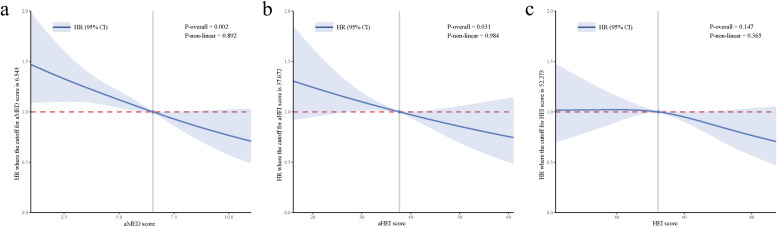
Association of anti-inflammatory dietary patterns with all-cause mortality risk (**a**) aMED with all-cause mortality; **b** AHEI-2010 with all-cause mortality; **c** HEI-2015 with all-cause mortality

Table 2 shows the association of three dietary scores with all-cause mortality in OSA patients. After adjusting demographic characteristics, higher aMED (HR = 0.60, 95%CI: 0.47 to 0.76) and HEI-2015 (HR = 0.72, 95%CI: 0.58 to 0.89) scores were associated with lower all-cause mortality risk in the elderly OSA patients. In fully adjusted model 2, higher scores of the aMED (HR = 0.61, 95%CI: 0.48 to 0.78) and HEI-2015 (HR = 0.75, 95%CI: 0.60 to 0.95) were associated with reduced risk of all-cause mortality. No association was observed between higher AHEI-2010 score and all-cause mortality risk in model 1 (HR = 0.79, 95%CI: 0.57 to 1.09) and model 2 (HR = 0.80, 95%CI: 0.57 to 1.12). Figure 2 shows that no nonlinear associations were observed between anti-inflammatory dietary patterns and all-cause mortality risk.

**Table 2:** Association of anti-inflammatory dietary scores and all-cause mortality among OSA patients

Variables	Unadjusted		Model 1		Model 2	
	HR (95% CI)	*P*	HR (95% CI)	*P*	HR (95% CI)	*P*
aMED score						
< 4	Ref		Ref		Ref	
4–6	0.81 (0.61–1.07)	0.137	0.72 (0.57–0.91)	0.007	0.70 (0.55–0.90)	0.005
≥ 6	0.64 (0.49–0.85)	0.002	0.60 (0.47–0.76)	< 0.001	0.61 (0.48–0.78)	< 0.001
AHEI-2010 score						
< 32	Ref		Ref		Ref	
32–40	0.87 (0.70–1.09)	0.221	0.89 (0.72–1.10)	0.272	0.80 (0.65–0.98)	0.032
≥ 40	0.81 (0.57–1.14)	0.218	0.79 (0.57–1.09)	0.150	0.80 (0.57–1.12)	0.190
HEI-2015 score						
< 47.11	Ref		Ref		Ref	
47.11–59.43	0.80 (0.67–0.97)	0.020	0.74 (0.61–0.89)	0.001	0.80 (0.66–0.98)	0.031
≥ 59.43	0.79 (0.61–1.04)	0.093	0.72 (0.58–0.89)	0.003	0.75 (0.60–0.95)	0.016

*HR* Hazard ratio, *CI* Confidence intervals, *Ref* Reference, *OSA* Obstructive sleep apnea, *aMED* Alternate Mediterranean Diet Score, *AHEI-2010* Alternate Healthy Eating Index-2010, *HEI-2015* Healthy Eating Index-2015

Unadjusted: Crude model

Model 1: Adjusted for demographic characteristics including age, race, poverty income ratio, marriage, and education

Model 2: Adjusted for age, race, poverty income ratio, marriage, education, body mass index, physical activity, hypertension, diabetes, cardiovascular disease, chronic kidney disease, chronic obstructive pulmonary disease, cancer, use of cardiovascular drugs, white blood cell, neutrophil, and C-reactive protein

### Subgroup analyses

**Fig. 3:**
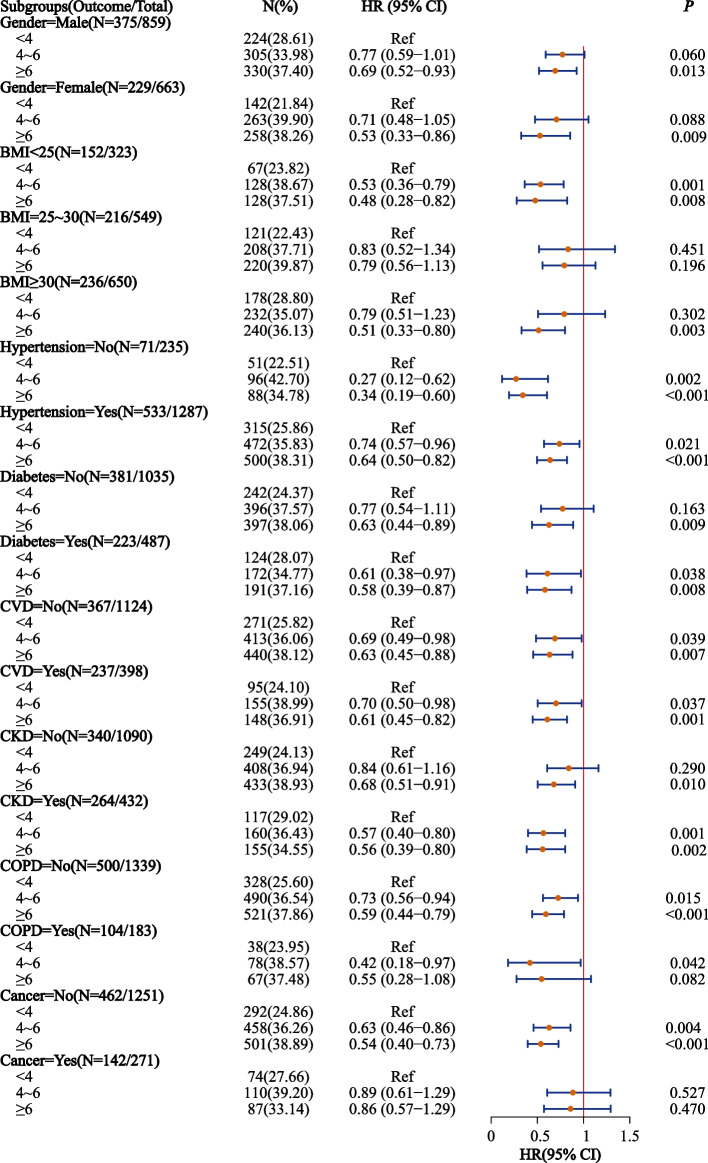
Association of aMED score and all-cause mortality in the elderly with OSA

**Fig. 4:**
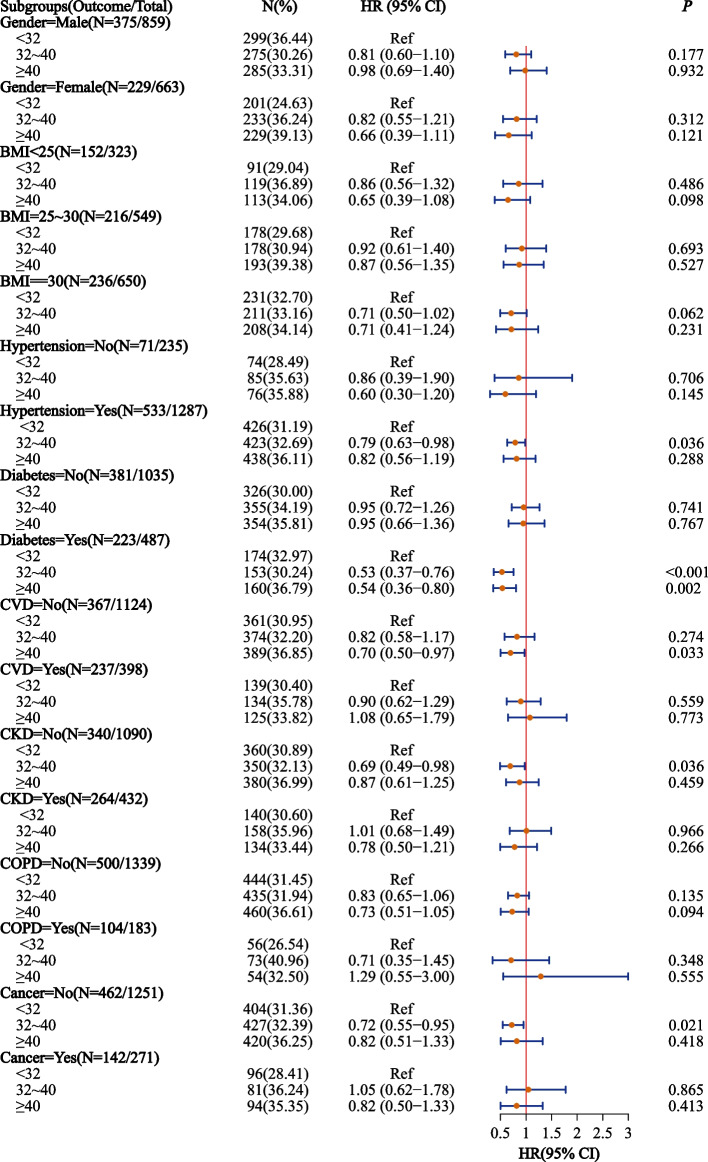
Association of AHEI-2010 score and all-cause mortality in the elderly with OSA

**Fig. 5:**
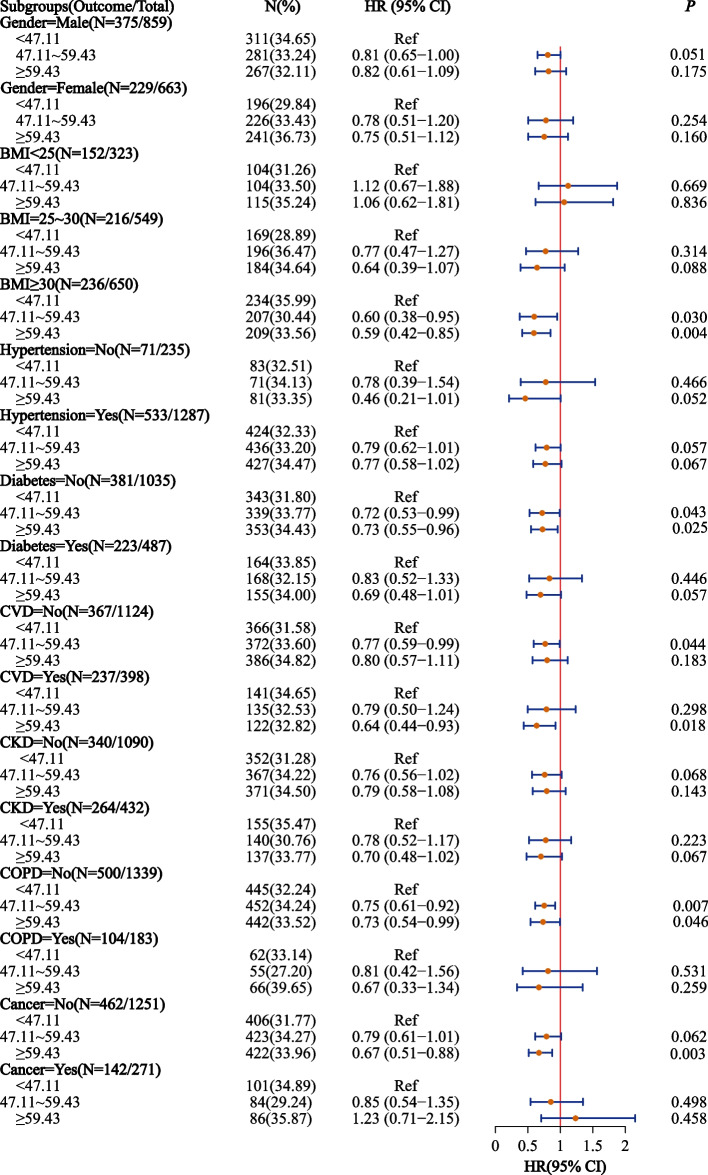
Association of HEI-2015 score and all-cause mortality in the elderly with OSA

The associations between anti-inflammatory patterns and all-cause mortality risk were further investigated in different subgroups. Higher aMED score were related to lower all-cause mortality risk in subgroups of male, female, BMI < 25, BMI ≥ 30, hypertension, non-hypertension, diabetes, non-diabetes, CVD, non-CVD, CKD, non-CKD, non-COPD, and non-cancer. Higher AHEI-2010 score was related to lower all-cause mortality risk in diabetes and non-CVD subgroups. Higher HEI-2015 score was related to lower all-cause mortality risk in BMI ≥ 30, non-diabetes, CVD, non-COPD, and non-cancer. Figures 3, 4 and 5 shows the associations between anti-inflammatory patterns and all-cause mortality risk in different subgroups.

### Components of aMED and all-cause mortality

From results above, aMED score is more applicable in OSA so an analysis was performed to investigate the association between components of aMED dietary pattern and all-cause mortality. Table 3 shows that higher vegetable (HR = 0.60, 95%CI: 0.48 to 0.76) and olive oil (HR = 0.67, 95%CI: 0.63 to 0.71) intake were associated with a lower all-cause mortality risk in the elderly with OSA. Moderate fish (HR = 0.60, 95%CI: 0.41 to 0.88) and dairy (HR = 0.76, 95%CI: 0.61 to 0.96) intake may also have potential benefits.

**Table 3:** Association of aMED components with all-cause mortality among OSA patients

Variables	Unadjusted		Model 1		Model 2	
	HR (95% CI)	*P*	HR (95% CI)	*P*	HR (95% CI)	*P*
aMED components						
Fruit						
< 1	Ref		Ref		Ref	
1–2	0.97 (0.75–1.25)	0.806	0.81 (0.61–1.06)	0.119	0.85 (0.64–1.13)	0.258
> 2	0.98 (0.69–1.39)	0.915	0.98 (0.66–1.44)	0.905	0.99 (0.66–1.46)	0.946
Vegetables						
< 0.5	Ref		Ref		Ref	
0.5–1	0.74 (0.52–1.06)	0.099	0.70 (0.50–0.98)	0.036	0.72 (0.52–0.99)	0.044
> 1	0.55 (0.41–0.75)	< 0.001	0.60 (0.48–0.75)	< 0.001	0.60 (0.48–0.76)	< 0.001
Legumes						
< 1	Ref		Ref		Ref	
1–2	0.79 (0.40–1.57)	0.498	0.95 (0.46–1.94)	0.881	1.16 (0.58–2.31)	0.669
> 2	0.75 (0.49–1.16)	0.201	0.84 (0.60–1.18)	0.321	0.66 (0.43–1.00)	0.053
Cereals						
< 1	Ref		Ref		Ref	
1–1.5	1.00 (0.69–1.45)	0.999	0.95 (0.63–1.44)	0.818	0.91 (0.57–1.45)	0.686
> 1.5	0.96 (0.75–1.22)	0.729	0.92 (0.76–1.12)	0.404	0.90 (0.73–1.11)	0.320
Fish						
< 1	Ref		Ref		Ref	
1–2.5	0.60 (0.40–0.91)	0.017	0.68 (0.44–1.04)	0.074	0.60 (0.41–0.88)	0.009
> 2.5	0.98 (0.70–1.37)	0.899	1.12 (0.90–1.41)	0.316	0.96 (0.75–1.23)	0.744
Meat						
< 1	Ref		Ref		Ref	
1–1.5	0.80 (0.47–1.35)	0.400	0.81 (0.48–1.37)	0.432	0.87 (0.54–1.42)	0.585
> 1.5	0.96 (0.78–1.18)	0.674	0.93 (0.75–1.14)	0.473	1.02 (0.82–1.26)	0.887
Dairy						
< 1	Ref		Ref		Ref	
1–1.5	0.71 (0.58–0.87)	0.001	0.65 (0.52–0.82)	< 0.001	0.76 (0.61–0.96)	0.019
> 1.5	0.88 (0.71–1.08)	0.225	0.88 (0.71–1.10)	0.272	0.94 (0.76–1.17)	0.597
Alcohol						
> 2	Ref		Ref		Ref	
< 1	1.91 (1.12–3.28)	0.018	1.27 (0.84–1.92)	0.257	1.12 (0.72–1.74)	0.604
1–2	1.29 (0.65–2.55)	0.471	0.90 (0.51–1.58)	0.723	0.77 (0.45–1.32)	0.345
Olive oil						
< 14	Ref		Ref		Ref	
14–28	0.00 (0.00–0.00)	< 0.001	0.00 (0.00–0.00)	< 0.001	0.00 (0.00–0.00)	< 0.001
> 28	0.65 (0.62–0.68)	< 0.001	0.66 (0.63–0.70)	< 0.001	0.67 (0.63–0.71)	< 0.001

*HR* Hazard ratio, *CI* Confidence intervals, *Ref* Reference, *aMED* Alternate Mediterranean Diet Score, *OSA* Obstructive sleep apnea

Unadjusted: Crude model

Model 1: Adjusted for demographic characteristics including age, race, poverty income ratio, marriage, and education

Model 2: Adjusted for age, race, poverty income ratio, marriage, education, body mass index, physical activity, hypertension, diabetes, cardiovascular disease, chronic kidney disease, chronic obstructive pulmonary disease, cancer, use of cardiovascular drugs, white blood cell, neutrophil, and C-reactive protein

## Discussion

Higher aMED adherence was associated with lower all-cause mortality risk in OSA patients. Similar results were found in HEI-2015. The association remained consistent across different subgroups on male, female, BMI < 25, BMI ≥ 30, hypertension, non-hypertension, diabetes, non-diabetes, CVD, non-CVD, CKD, non-CKD, non-COPD, and non-cancer for aMED. Encourage the adoption of healthy dietary patterns can be considered a strategy for managing OSA.

Our findings on the association between high adherence of aMED and lower all-cause mortality risk in the elderly with OSA were consistent with previous study in the general population [19]. Low aMED score was related to adverse health outcomes including CVD [15], pregnancy outcomes [32], cancer [33] and mortality [19]. The Mediterranean diet, characterized by a high intake of fruits, vegetables, and whole grains rich in essential nutrients and fiber, has been linked to antioxidant and anti-inflammatory effects, which may help alleviate systemic inflammation in OSA patients [34]. The diet's high fiber content in vegetables promotes gastrointestinal health, regulates blood sugar levels, and aids in weight management, thus reducing the risk of chronic diseases associated with obesity and metabolic dysfunction [35]. Furthermore, the primary fat source in the aMED diet, olive oil, is known for its high monounsaturated fatty acid (MUFA) content, which has anti-inflammatory properties and a positive impact on lipid levels, contributing to a reduced risk of CVD [36]. Additionally, the phenolic compounds in extra-virgin olive oil exerts potent antioxidant effects, protecting against oxidative stress-induced cellular damage [37].

Our results were also consistent with previous studies that reported inverse associations between adherence of HEI-2015 and all-cause mortality [16, 38]. Higher HEI-2015 scores had a reduction in risk of all-cause, CVD, and cancer mortality [39]. And an inverse relationship was observed between HEI-2015 score and plasma biomarkers of chronic inflammation [40]. Similar to aMED, nutrients in fruits, vegetables and whole grain have anti-inflammatory and antioxidants properties. The intake of l dairy recommended by HEI-2015 was a source of essential nutrients like calcium, vitamin D and probiotics [41]. These components contributed to bone health, support immune function, and promote overall well-being in OSA [42]. HEI-2015 limited intake of added sugars to lower overall calorie consumption, and excessive added sugars intake has been linked to obesity, diabetes and CVD [43]. HEI-2015 limited intake of saturated fats and urges the substitution with healthier fats, so adhering to HEI-2015 helps maintain a favorable lipid profile in patients with OSA [44]. Reducing sodium intake, contribute to maintain blood pressure within health ranges, and potential have lower hypertension and complications risk in OSA [45].

The association between anti-inflammatory dietary patterns and all-cause mortality can be contributed to several potential mechanisms. First, anti-inflammatory dietaries are rich in fruits, vegetables, whole grains, and healthy fats, which are abundant sources of antioxidants and polyphenols [46]. These bioactive compounds have been shown to possess anti-inflammatory properties and help reduce systemic inflammation, a key underlying factor in the development and progression of chronic disease [47]. Second, anti-inflammatory diets often include foods rich in omega-3 fatty acids, such as fatty fish, walnuts, and flaxseeds. Omega-3 fatty acids have been shown to exert anti-inflammatory effects by modulating immune response and reducing the production of pro-inflammatory cytokines [48, 49]. In addition, omega-3 fatty acids were associated with improved cardiovascular health [50]. Furthermore, limiting the intake of pro-inflammatory foods, such as processed meats, refined grains, and added sugars. These foods have been shown to promote inflammation and increase the risk of chronic disease, including cardiovascular disease and cancer [51, 52].

The findings indicated that adopting an anti-inflammatory dietary pattern may have better health outcomes in OSA. Mediterranean diet pattern and dietary pattern based on the 2015 to 2020 Dietary Guidelines for Americans were recommended. Healthy dietary educations should be conducted by health workers, raising awareness of the future benefits with healthy food choices in population.

The discrepancy observed between subgroups and the overall findings may be explained by several reasons. Firstly, the sample size might be insufficient to identify a significant association between anti-inflammatory dietary patterns and all-cause mortality in specific subgroups. Subgroup analyses inherently possess less statistical power than the overall participants, making the detection of significant associations in smaller subgroups more challenging. Secondly, there may be confounding variables present in subgroups that were not fully adjusted in the analysis. Thirdly, interactions between anti-inflammatory dietary patterns and the specific characteristics of the subgroups might not be considered. For instance, the impact of aMED on all-cause mortality may be influenced by variables such as BMI, COPD, or cancer, resulting in different associations in these subgroups.

There were several strengths in our study, including a relatively long follow-up period, and a large cohort with sufficient power to detect statistically significant associations. And our findings were not solely influenced by the large sample size. However, some limitations should also be acknowledged. The dietary assessment relied on self-reported data, which may lead to recall bias. Besides, dietary intake data were only obtained at baseline, which may not reflect long-term dietary patterns and changes during the follow-up. Third, the covariates utilized for analysis were solely those available within the database, and other potential covariates unmeasured were hard to adjust. Finally, our study only investigated in older adults with OSA and may not be generalizable to other age groups or individuals without OSA. Additionally, the data from the NHANES database may not account for regional or cultural variations in dietary patterns.

## Conclusion

Higher adherence to the aMED and the HEI-2015 was associated with lower all-cause mortality risk in the elderly with OSA. More research is need to investigate the mechanism of this association. Future interventions in the elderly with OSA could considering adopting dietary patterns based on aMED or HEI-2015.

## Data Availability

The datasets generated and/or analyzed during the current study are available in the NHANES database, https://www.cdc.gov/nchs/nhanes.

## Abbreviations

OSA: Obstructive sleep apnea
CV: Cardiovascular
aMED: Alternate Mediterranean Diet Score
HEI-2015: Healthy Eating Index-2015
AHEI-2010: Alternate Healthy Eating Index-2010
NHANES: National Health and Nutrition Examinations Survey
NCHS: National Center for Health Statistics
PIR: Poverty income ratio
CVD: Cardiovascular disease
CKD: Chronic kidney disease
COPD: Chronic obstructive pulmonary disease
HbA1c: Hemoglobin A1c
S.E: Standard error
HR: Hazard ratio
CI: Confidence interval
